# NMR-based metabolomics for simultaneously evaluating multiple determinants of primary beef quality in Japanese Black cattle

**DOI:** 10.1038/s41598-017-01272-8

**Published:** 2017-05-02

**Authors:** Yoshinori Kodani, Takuya Miyakawa, Tomohiko Komatsu, Masaru Tanokura

**Affiliations:** 1 Department of Applied Biological Chemistry, Graduate School of Agricultural and Life Sciences, The University of Tokyo, 1-1-1 Yayoi, Bunkyo-ku, Tokyo 113-8657 Japan; 2 Yamagata Prefectural Animal Industrial Institute, Agricultural Research Center, Shinjo, Yamagata 996-0041 Japan

## Abstract

Analytical methodologies to comprehensively evaluate beef quality are increasingly needed to accelerate improvement in both breeding and post-mortem processing. Consumer palatability towards beef is generally attributed to tenderness, flavor, and/or juiciness. These primary qualities are modified by post-mortem aging and the crude content and fatty acid composition of intramuscular fat. In this study, we report a nuclear magnetic resonance (NMR)-based metabolic profiles of Japanese Black cattle to evaluate the compositional attributes of intramuscular fat and the long-term post-mortem aging. The unsaturation degree of triacylglycerol was estimated by the ^1^H NMR spectra and was correlated with the content ratio of unsaturated fatty acids (*R* ^2^ = 0.944) and the melting point of intramuscular fat (*R* ^2^ = 0.871). NMR-detected profiles of water-soluble metabolites revealed overall metabolic change (*R* ^2^ = 0.951) and several metabolites (*R* ^2^ > 0.818) linearly correlated with long-term aging duration, which can be used to evaluate the aging rate and aging duration of beef. This approach also provided the pH profile during aging, which is related to the water-holding capacity of beef. Thus, NMR-based metabolomics has the potential to evaluate multiple parameters related to the beef qualities of Japanese Black cattle.

## Introduction

Beef is among the edible meats with high demand in many countries. Consumer palatability toward beef is generally attributed to tenderness, flavor, and/or juiciness^1^. The primary beef qualities are improved by post-mortem aging due to proteolysis within the muscle tissue structures, such as myofibrillar proteins, over time^1–4^. Beef metabolites are changed during aging, e.g., amino acid production by proteolytic degradation. Water-soluble metabolites, such as sugars and amino acids, contribute to the production of beef flavor with heat treatment through the Maillard reaction^5^. In Japanese Black cattle, beef quality has been improved, based on the bovine marbling score (BMS), through genetic selection. In addition to post-mortem aging, the crude fat and the fatty acid compositions attribute to the tenderness, flavor, and/or juiciness, which affect the palatability of Japanese Black cattle^6–8^. Among the fatty acids composing the intramuscular fat in beef, the content of unsaturated fatty acids is most strongly related to beef quality parameters, such as the strength of the aroma and flavor^9^.

There is an increasing need for holistic analytical techniques capable of rapidly assessing beef quality parameters. Impedance measurement is an analytical technique used for a broad range of beef quality parameters, such as fat content, tenderness, ultimate pH value and aging^10^. Spectroscopic techniques, such as near infrared reflectance spectroscopy (NIRS) and Raman spectroscopy, have potential use for the evaluation of tenderness and water-holding capacity^10^. These techniques also provide structural information on the changes of myofibrillar proteins, water and lipids. As non-destructive techniques, ultrasound and color images were applied to measure the texture and the content of intramuscular fat^11^. Several analytical techniques, such as gas-chromatography or liquid-chromatography coupled with mass spectrometry (GC-MS or LC-MS) and nuclear magnetic resonance (NMR) spectroscopy, are required to measure diverse types of low molecular weight metabolites, such as sugars, amino acids, nucleotides, lipids and aromatic compounds^12^. These techniques are often combined with chemometric analysis to evaluate the beef quality parameters^13^. NMR is a high-throughput analytical technique that enables simultaneous and reproducible detection of a large number of metabolites and systematic metabolic changes in biological samples^14–18^. NMR spectroscopy is used to obtain metabolic profiles of food samples without complicated manipulation^19–23^. Several NMR studies have been reported on the metabolic profiles of beef, which are related to conservation^24^, post-mortem aging^25, 26^, irradiation doses^27^, and geographic origins^28^.

In the present study, we aimed to identify NMR-detected metabolite indicators to simultaneously evaluate multiple determinants of primary beef quality, including the composition of intramuscular fat and the progression of the aging process, in advanced marbling meat from Japanese Black cattle. Although long-term aged beef (over 4 weeks) is often marketed as quality-modified meat of heifers and delivered cows, the metabolic profiles remain unknown in the long-term aging process. We analyzed the long-term changes in metabolites in wet-aging beef samples, which were strictly controlled in a laboratory. In addition, our approach evaluated several indicators related to beef quality, such as the composition of intramuscular fat.

## Results and Discussion

### Compositional analysis of sirloin lean tissues using ^1^H NMR spectra

**Figure 1:**
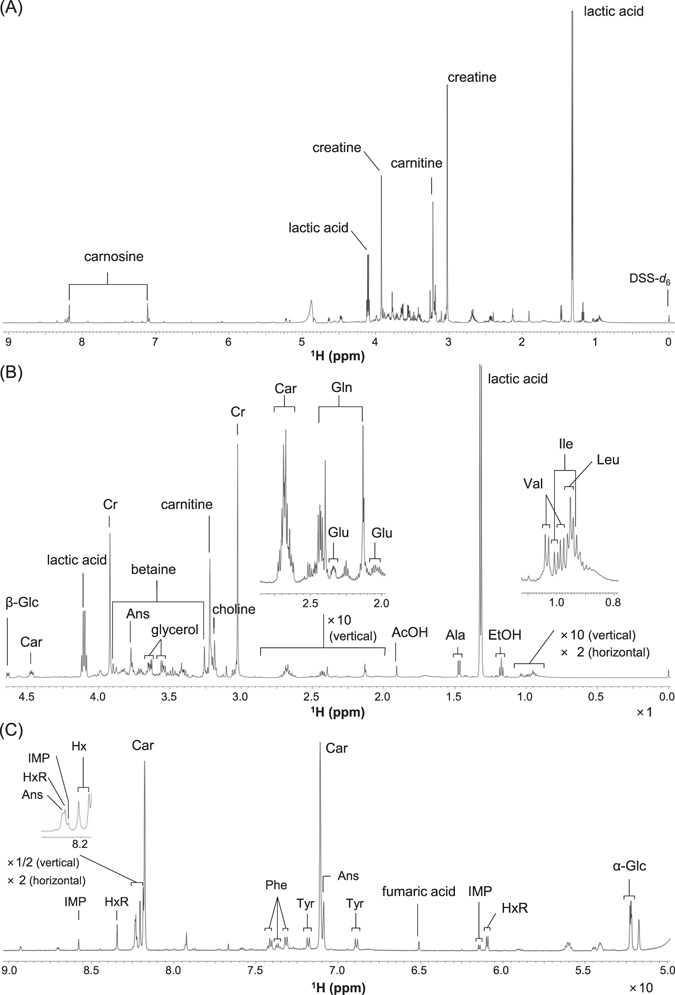
^1^H NMR spectra of the D_2_O extract of a sirloin sample from a brand A Black heifer (2-week aging). Assigned signals are labeled in (**A**) full spectrum, (**B**) high-field region, and (**C**) low-field region. AcOH, acetic acid; Ala, alanine; Ans, anserine; Car, carnosine; Cr, creatine; EtOH, ethanol; α-Glc, α-D-glucose; β-Glc, β-D-glucose; Gln, glutamine; Glu, glutamic acid; Hx, hypoxanthine; HxR, inosine; Ile, isoleucine; IMP, inosine monophosphate; Leu, leucine; Phe, phenylalanine; Tyr, tyrosine; and Val, valine.

^1^H NMR spectra were obtained from the D_2_O extracts of sirloin samples that are standard edible part in the experiments. Figure 1 shows a representative spectrum of a sirloin sample. Detected signals were derived from various water-soluble compounds, which were mainly extracted from sirloin lean tissues. Based on the 2D NMR analyses and the spiking experiments, 25 compounds were identified in the D_2_O extracts of the sirloin samples (Supplementary Table S1).

Among the identified compounds, 13 compounds were amino acids (alanine, anserine, carnitine, carnosine, creatine, glutamic acid, glutamine, glycine, isoleucine, leucine, phenylalanine, tyrosine, and valine). Generally, amino acids contribute to various gustatory sensations^29^. For example, alanine, glutamine and glycine contribute to sweetness; isoleucine, leucine, phenylalanine, tyrosine and valine are associated with bitterness; and a glutamate is typically associated with umami^29^. Creatine is stored in muscle as creatine phosphate, which is used to resynthesize adenosine 5′-triphosphate (ATP) from adenosine 5′-diphosphate (ADP). Creatine is also converted to a brothy taste modifier, *N*-(4-methyl-5-oxo-1-imidazolin-2-yl)sarcosine, through an aminocarbonyl reaction with methylglyoxal during heat treatment^30^. Anserine and carnosine are classified as imidazole dipeptides. They are contained in large amounts in vertebrate muscles and prevent a decrease in pH by lactic acid accumulation during exercise^31^. Carnitine is a characteristic compound in beef; its content is higher in beef than in the meat of chicken and pig^32^.

α-Glucose and β-glucose were detected in a molar ratio of 8:9 in the D_2_O extracts of sirloin samples using ^1^H NMR spectroscopy. Although molar ratio of α-glucose to β-glucose is generally known to be 37:63 at pH 7, it is affected by solution conditions such as pH value^33^. Glucose is generally produced from glycogen stored in muscle. Inosine (HxR), inosine 5′-monophosphate (IMP) and hypoxanthine (Hx) were identified as purine derivatives, which are often produced by ATP hydrolysis during the aging process of beef samples^12^. IMP is a typical compound with an umami taste^34^. Acetic acid, fumaric acid and lactic acid were detectable organic acids in the D_2_O extracts of sirloin samples. Acetic acid is a volatile fatty acid (VFA) that is produced through cellulose degradation mediated by rumen bacteria. Fumaric acid is often used as a supplement to elevate the utilization efficiency of forage nutrition and the VFA concentration of fattening cattle^35^. Lactic acid showed the highest signal intensity among the signals of other compounds. Ethanol, glycerol, betaine and choline were also identified in the D_2_O extracts of sirloin samples. Betaine is biologically synthesized as a metabolite of choline and is also used as a food additive because of its taste modification effects.

Among the compounds identified in the D_2_O extracts, 21 compounds were quantified by comparing the integral values of well-separated signals (asterisks in Supplementary Table S1) with that of the DSS-*d*_6_ signal (0 ppm). Table 1 summarizes the quantitative value of each compound. In accordance with the signal intensities, lactic acid was the most abundant compound in the D_2_O extracts. The amount was 23- and 63-fold those of acetic acid and fumaric acid, respectively, indicating that lactic acid is a major source of sour taste in beef. After slaughtering, lactic acid is dramatically increased through glycogen degradation and the growth of lactic acid bacteria under the anaerobic conditions of wet-aging^36^, affecting the ultimate pH, which contributes to meat tenderness and water-holding capacity^37^.

**Table 1:** Contents of the Compounds Detected in the D_2_O Extracts.

Compounds	Content (*μ*mol/g meat^a^)
*Amino acids*	
alanine	0.203 ± 0.036^b^
carnitine	0.377 ± 0.060
carnosine	0.567 ± 0.134
creatine	1.28 ± 0.24
glutamic acid	0.092 ± 0.012
glutamine	0.360 ± 0.083
isoleucine	0.008 ± 0.003
leucine	0.218 ± 0.038
phenylalanine	0.042 ± 0.006
tyrosine	0.036 ± 0.005
valine	0.070 ± 0.013
*Sugars*	
α-glucose	0.159 ± 0.019
β-glucose	0.180 ± 0.030
*Purine derivatives*	
inosine	0.057 ± 0.013
inosine monophosphate	0.045 ± 0.014
*Organic acids*	
acetic acid	0.115 ± 0.018
fumaric acid	0.043 ± 0.006
lactic acid	2.70 ± 0.47
*Alcohol*	
ethanol	0.056 ± 0.013
glycerol	0.460 ± 0.068
*Other compounds*	
betaine	0.089 ± 0.022

^a^Sirloin from brand A Black heifers (2-week aging).

^b^Mean ± standard deviation (n = 5).

### NMR-based compositional analysis of sirloin intramuscular fat

**Figure 2:**
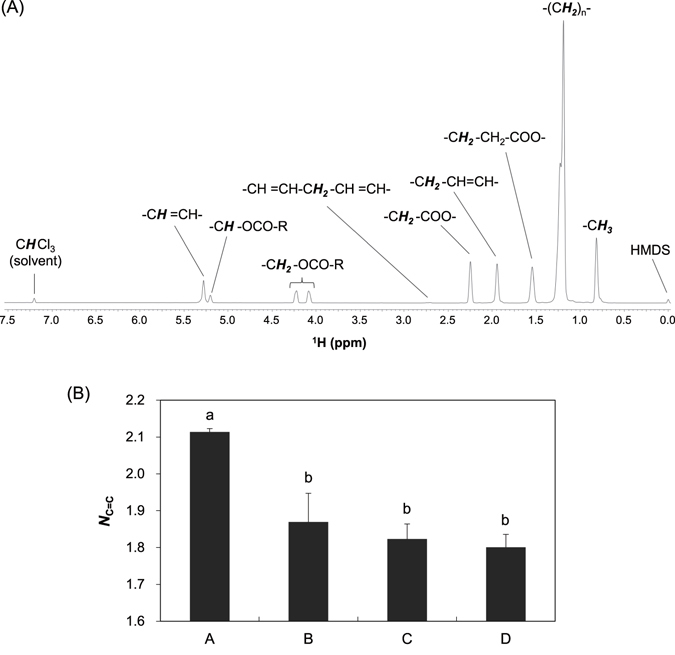
Triacylglycerol (TAG) and its unsaturation degree (*N*_C=C_) in the sirloin of Japanese Black cattle. (**A**) ^1^H NMR spectra of the CDCl_3_ extract of a sirloin sample after 2-week aging. Each signal is assigned to the proton represented by the italic and bold style (***H***) in the partial structural formula of TAG. (**B**) The *N*_C=C_ values of the sirloin samples from four different brands. The data are presented as the mean ± standard error (SE; *n* = 5). Means with different letters (a and b) are significantly different at *p* < 0.05.

Figure 2 shows a representative spectrum of the CDCl_3_ extract of a sirloin sample. The spectrum was consistent with the typical signal pattern of triacylglycerol (TAG), in which a glycerol backbone forms ester bonds with three fatty acid moieties. The signal pattern characterizing TAG was observed as triplicate signals of the glycerol backbone at 4.09 and 4.22 ppm (-C***H***_***2***_-OCO-R) and 5.20 ppm (-C***H***-OCO-R). There was no signal from mono- and diacylglycerol in the ^1^H NMR spectra of the CDCl_3_ extract.

Although TAG can be composed of various types of fatty acids, only 7 major signals derived from fatty acid moieties were observed in the ^1^H NMR spectra (Fig. 2A). These signals were shifted in the spectra based on the relative position of double bond(s) and/or ester linkage. The signal at 5.28 ppm was derived from the protons directly attached to the carbon forming a double bond, which provided the double bond content in TAG as its integral value. The TAG content in the CDCl_3_ extract was represented as the integral of the signal at 4.09 or 4.22 ppm. Therefore, we estimated the unsaturation degree of TAG (*N*_C=C_) using the following equation (1) and the integral values of the signals at 5.28 ([CH=CH]) and 4.22 ppm (or 4.09 ppm, [TAG]).

1 $${N}_{{\rm{C}}={\rm{C}}}=[{\rm{CH}}=\mathrm{CH}]/[\mathrm{TAG}]$$

The *N*_C=C_ values of 20 sirloin samples (5 samples from each of the four brands) were calculated in the range from 1.69 to 2.14. Figure 2B shows the *N*_C=C_ values of sirloin samples derived from different brands of Black heifers. The averaged *N*_C=C_ values were 2.11, 1.87, 1.82, and 1.80 in the sirloin samples of brands A, B, C and D, respectively. Brand A had a significantly higher *N*_C=C_ than the other brands, suggesting that brand A may be characterized by a high ratio of unsaturated fatty acids although the fatty acid compositions are influenced by the dietary backgrounds/production systems of the animals.

### Correlation of NMR-detected *N*_C=C_ values with intramuscular fat quality

**Figure 3:**
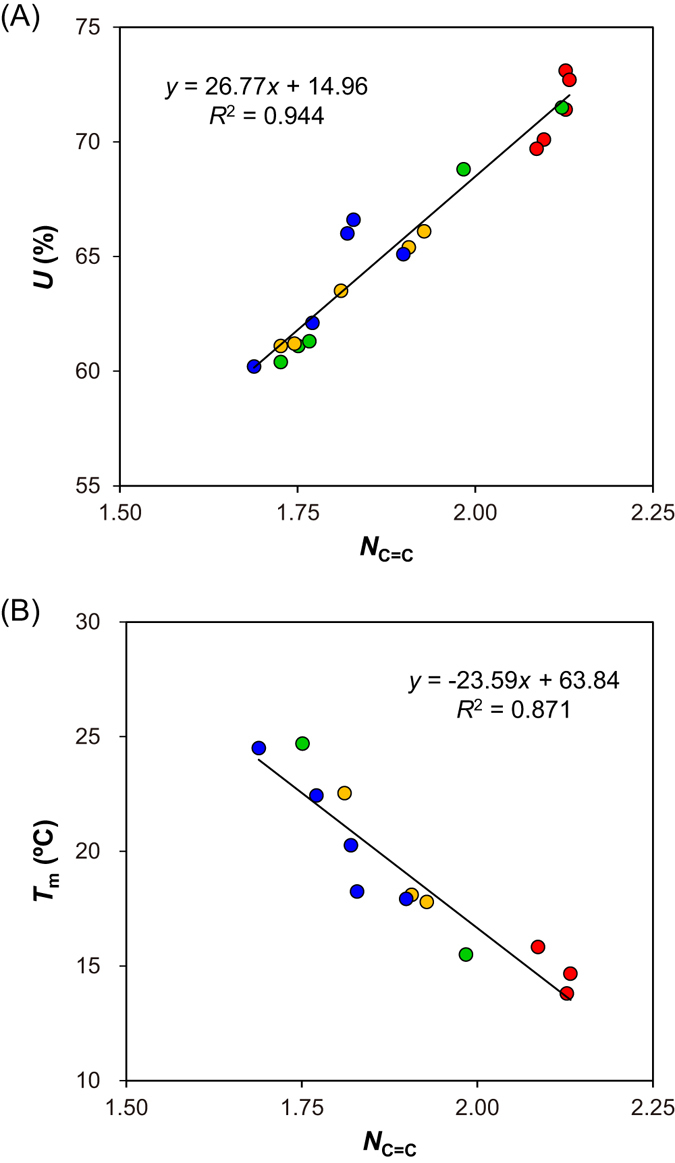
Correlation of the unsaturation degree of TAG (*N*_C=C_) with (**A**) the ratio of unsaturated fatty acids (*U*) and (**B**) the melting point (*T*_m_) of the sirloin samples. The plots are color-coded according to brand: A, red; B, green; C, yellow; and D, blue.

The fatty acids in the intramuscular fat of beef were measured using GC-FID. We analyzed 7 major fatty acids in the intramuscular fat of sirloin using GC-FID and compared the total ratio of unsaturated fatty acids with the NMR-detected *N*_C=C_ value. The ratio of each fatty acid was summarized as a weight percent in Table 2. Oleic acid (C18:1, *cis*-9) showed the highest ratio (>50 wt%) among the observed fatty acids. The ratios of other fatty acids decreased in the following order: palmitic acid (C16:0) > stearic acid (C18:0) > palmitoleic acid (C16:1, *cis*-9) > linoleic acid (C18:2, *cis*-9,12) > myristic acid (C14:0) > myristoleic acid (C14:1, *cis*-9). However, palmitoleic acid (C16:1, *cis*-9) was found at a higher content than stearic acid (C18:0) only in the sirloin samples of brand A, which may be the cause of the higher *N*_C=C_ value of brand A. The total ratio of unsaturated fatty acids ranged from 60.2 wt% to 73.1 wt%, which was calculated by summation of the ratio of myristoleic acid (C14:1, *cis*-9), palmitoleic acid (C16:1, *cis*-9), oleic acid (C18:1, *cis*-9), and linoleic acid (C18:2, *cis*-9,12). The NMR-detected *N*_C=C_ values were plotted against the total ratio of unsaturated fatty acids (Fig. 3A), which showed a good correlation between these two evaluated values (*R* ^2^ = 0.944). The results suggest that the total ratio of unsaturated fatty acids, which attribute to the tenderness, flavor, and/or juiciness^6–8^, can be estimated using the NMR-detected *N*_C=C_ value.

**Table 2:** Fatty Acid Composition of the Sirloin Derived from Japanese Black Cattle.

Brand	Ratio (wt%)								MW^b^	*N*_C=C_
	C14:0	C14:1	C16:0	C16:1	C18:0	C18:1	C18:2	UFA^a^		
A	2.5	2.0	21.8	8.1	5.5	56.2	3.9	70.1	851.1	2.10
	2.0	1.6	18.7	7.0	6.3	60.3	4.2	73.1	856.3	2.13
	2.1	1.6	20.5	6.8	5.9	58.3	4.7	71.4	854.6	2.13
	2.0	1.8	19.7	7.8	5.6	59.0	4.0	72.7	854.2	2.13
	1.8	1.1	21.0	6.0	7.5	59.4	3.1	69.7	856.6	2.09
B	2.9	0.9	26.8	5.5	9.2	51.8	2.8	61.1	850.9	1.75
	1.9	1.2	19.2	5.5	7.4	60.4	4.4	71.5	858.2	2.12
	2.3	1.4	21.3	6.3	7.6	57.5	3.6	68.8	854.7	1.98
	2.9	0.9	26.6	4.9	10.1	52.2	2.4	60.4	851.8	1.73
	3.1	1.5	27.4	6.2	8.2	50.9	2.6	61.3	848.3	1.77
C	3.6	1.1	27.6	6.5	7.8	50.2	3.3	61.1	847.8	1.73
	2.8	1.5	25.4	6.1	8.3	52.9	3.0	63.5	850.5	1.81
	3.3	1.7	26.6	6.4	8.9	51.1	2.0	61.2	848.1	1.75
	2.2	1.1	22.4	5.5	9.3	56.5	3.0	66.1	855.3	1.93
	2.3	1.0	23.9	5.8	8.4	55.4	3.1	65.4	853.8	1.91
D	1.5	0.7	23.6	4.9	8.9	57.8	2.6	66.0	857.2	1.82
	1.9	0.9	21.2	4.9	10.3	57.5	3.2	66.6	857.8	1.83
	2.4	1.1	27.4	5.2	10.1	51.4	2.4	60.2	851.4	1.69
	2.5	1.2	25.3	5.1	10.2	52.8	3.1	62.1	852.9	1.77
	2.2	1.1	23.5	5.5	9.2	55.4	2.9	65.1	854.4	1.90

^a^Total ratio of unsaturated fatty acids.

^b^Averaged molecular weight of triacylglycerol (TAG).

In general, the melting point of each fatty acid is related to its structure, including the numbers of double bonds and carbon atoms. We further analyzed the correlation between the NMR-detected *N*_C=C_ and melting point. Thirteen sirloin samples were chosen to cover the broad range of the *N*_C=C_ value: 3 brand A, 2 brand B, 3 brand C, and 5 brand D. In Fig. 3B, the NMR-detected *N*_C=C_ values were plotted against the melting points of 13 sirloin samples. The plots showed a good correlation between the *N*_C=C_ value and the melting point (*R* ^2^ = 0.871). The melting point was decreased in association with the increased *N*_C=C_ value. The melting point is considered to be one of indicators of beef quality in Japanese Black cattle because it is related to tenderness and juiciness through the solubility of intramuscular fat in the mouth. The NMR-detected *N*_C=C_ value provides a useful and simple method to evaluate the melting point of TAG in various food materials, such as beef, regardless of the melting point range.

Among the major indicators of edible fat and oil, the iodine value is widely used to evaluate the chemical and physicochemical properties, such as oxidative stability and fat firmness^38^. In a pure lipid compound, the iodine value (IV) is calculated based on its molecular weight (MW) and unsaturation degree (*N*_C=C_) according to the following equation (2) ^39^.

2 $${\rm{IV}}[{\rm{g}}]=100[{\rm{g}}]/{{\rm{MW}}}_{{\rm{lipid}}}\times {{\rm{MW}}}_{{\rm{I}}}\times {N}_{{\rm{C}}={\rm{C}}}$$

MW_lipid_ and MW_I_ represent the molecular weight of the lipid and iodine (253.8), respectively. Although the intramuscular fat of beef is composed of various types of lipids, the NMR-detected *N*_C=C_ of TAG was used to calculate the iodine value of the sirloin samples in the present study. In addition, the MW of TAG in the samples was estimated based on the ratio and average MW of each fatty acid (Table 2) and was in the range from 847.9 to 858.2. The standard deviation was approximately 1.2% of the averaged MW of all samples (853.3), which is much lower than the standard deviation of *N*_C=C_ (~23%). Therefore, it is possible to use the averaged MW to calculate the iodine value based on the NMR-detected *N*_C=C_ value.

### NMR-based evaluation of the long-term aging process

**Figure 4:**
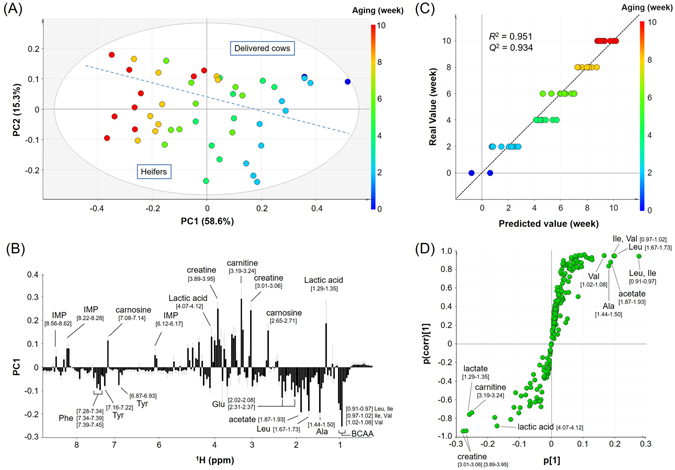
Multivariate data analysis of the ^1^H NMR spectra of the D_2_O extracts of ribeye samples with different aging duration. Ribeye samples are derived from heifers and delivered cows. (**A**) PCA score plot of PC1 versus PC2. The plots are color-coded according to aging duration (week): 0, blue; 2, cyan; 4, turquoise; 6, light green; 8, yellow; and 10, red. The heifer samples are positioned separately from the delivered cow on the score plot, and the individual regions are divided by a dashed line. (**B**) Loading plot for PC1 of the score plot shown in panel A. Buckets with high loading values are labeled by the assigned compound names. The square brackets represent the chemical shift range of each spectral bucket. (**C**) OPLS regression model for the prediction of aging duration. Predicted values are derived from the OPLS model of the ^1^H NMR spectra. The plots are color-coded according to aging duration (week): 0, blue; 2, cyan; 4, turquoise; 6, light green; 8, yellow; and 10, red. (**D**) S plot for the OPLS regression model shown in panel C. Buckets with high absolute values of p[1] and p(corr)[1] are labeled with the assigned compound names. The square brackets represent the chemical shift range of each spectral bucket.

^1^H NMR spectra were obtained from the D_2_O and CDCl_3_ extracts of ribeye samples after different aging duration. Beef ribeye is a major edible part that is generally subjected to post-mortem aging. We chose both heifers and delivered cows to generalize the evaluation model of long-term post-mortem aging. The data set of spectral buckets was processed by multivariate data analysis to determine the overall compositional changes during aging. Figure 4A shows the PCA score plot of the D_2_O extracts of ribeye samples from 5 heifers and 4 delivered cows. All samples were distributed in the 95% confidence interval of the score plot with high statistical values of *R* ^2^ *x* (58.6% for PC1 and 15.3% for PC2). As aging proceeded, the plots shifted from positive to negative along the PC1 axis, suggesting that the compounds contributing to PC1 characterize the aging process. The loading plot of the PC1 axis (Fig. 4B) indicated that after longer aging duration, the ribeye samples contained higher amounts of acetic acid, alanine, glutamic acid, isoleucine, leucine, phenylalanine, tyrosine and valine, and lower amounts of carnitine, carnosine, creatine, IMP and lactic acid. PC2 reflected the differences between heifers and delivered cows, including the age-related influence. Based on the loading plot of the PC2 axis, the amount of alanine, anserine, betaine, fumaric acid and glutamine in heifers is higher than that in delivered cows (Supplementary Fig. S1).

The PCA score plot was also investigated for the CDCl_3_ extracts of ribeye samples (Supplementary Fig. S2). The *R* ^2^ *x* values were 56.4% for PC1 and 33.2% for PC2. In contrast to the D_2_O extracts, the distribution of samples did not provide information related to differences in aging duration. All heifer samples were separated from the delivered cow samples in the region of negative PC1 and positive PC2. The total fat contents were 38.6 ± 5.9 wt% and 17.3 ± 3.6 wt% in the ribeye samples from heifers and delivered cows, respectively. These results suggest that the TAG composition may be strongly influenced by the fattening process rather than the aging process.

**Figure 5:**
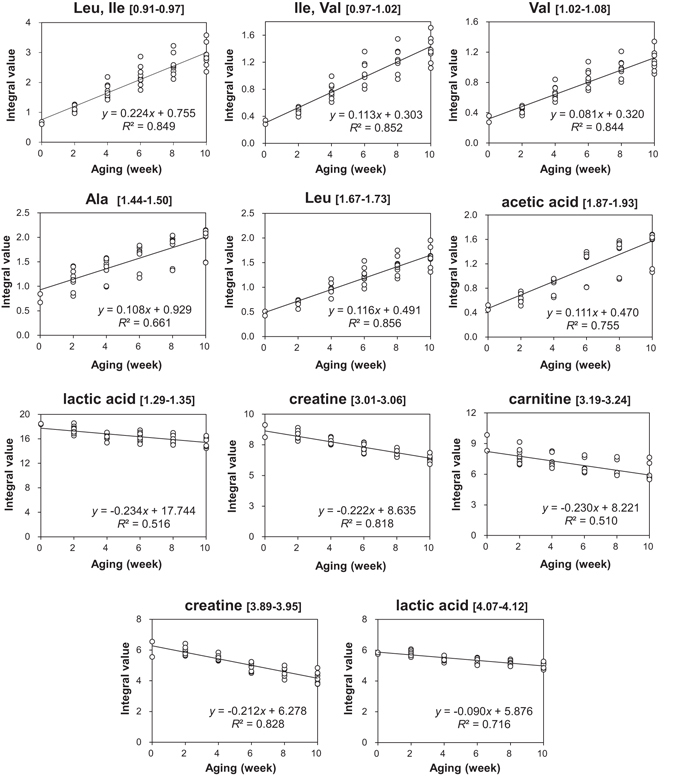
Changes in the contents of aging-related compounds, which are given as the integral values of individual buckets. The square brackets represent the chemical shift range of each spectral bucket, which is labeled by the assigned compound name.

According to the PCA results, we built an OPLS regression model to predict aging duration using the ^1^H NMR spectra of the D_2_O extracts. As shown in Fig. 4C, the actual values of aging duration were well correlated with the aging duration predicted by the NMR data. The goodness of fit (*R* ^2^) and the predictability (*Q* ^2^) were 0.951 and 0.934, respectively. The S plot for the OPLS regression model was used to evaluate the compounds related to aging (Fig. 4D). As suggested by the loading plot (Fig. 4B), aging duration was positively correlated with acetic acid, alanine, isoleucine, leucine and valine. Carnitine, creatine, and lactic acid showed a negative correlation with aging duration. To evaluate whether the contents of these compounds were linearly changed by an increased aging duration, the integral value of each bucket was further plotted against aging duration. As shown in Fig. 5, acetic acid, alanine, isoleucine, leucine and valine increased with aging. Leucine (a spectral bucket of 1.67‒1.73 ppm) showed the strongest positive correlation with aging duration (*R* ^2^ = 0.856). In contrast, creatine (a spectral bucket of 3.89‒3.95 ppm) had the strongest negative correlation with aging duration (*R* ^2^ = 0.828) among the other compounds, including carnitine and lactic acid.

The *K* value is a robust indicator of freshness and aging and is defined by the following equation (3) according to the content of nucleotides ([ATP], [ADP], [AMP] and [IMP]), inosine ([HxR]) and hypoxanthine ([Hx])^40^.

3 $$K=([{\rm{HxR}}]+[{\rm{Hx}}])/([{\rm{ATP}}]+[{\rm{ADP}}]+[{\rm{AMP}}]+[{\rm{IMP}}]+[{\rm{HxR}}]+[{\rm{Hx}}])$$

**Figure 6:**
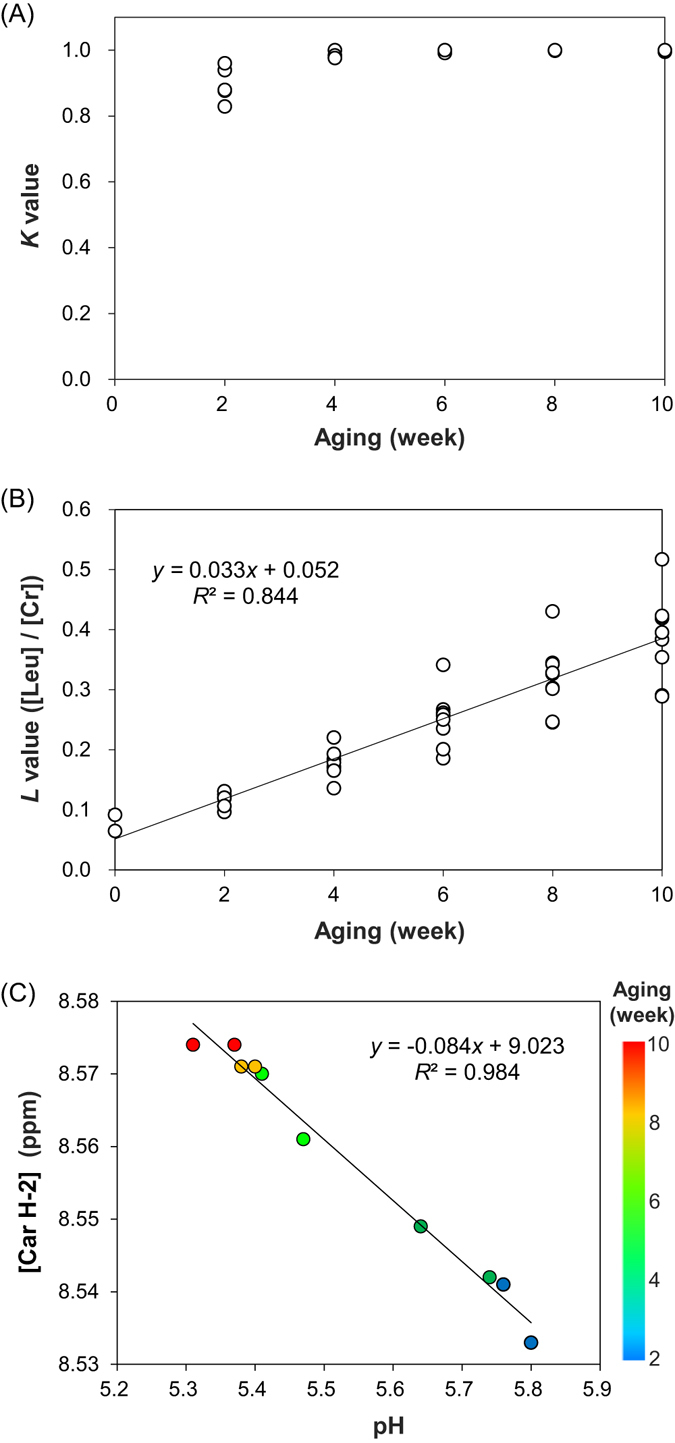
Indicators of aging progression. Correlation of aging duration with (**A**) *K* value and (**B**) *L* value, represented by the ratio of leucine ([Leu]) to creatine ([Cr]). (**C**) Correlation between pH and [Car H-2], represented by the chemical shift of the ^1^H signal at C-2 of the imidazole ring of carnosine (Car). The [Car H-2] value in the observed pH range shows a linear correlation with pH, although overall pH profile of [Car H-2] is sigmoidal^42^. The plots are color-coded according to aging duration (week): 2, blue; 4, green; 6, light green; 8, yellow; and 10, red.

The ^1^H NMR spectra provided the total content of ATP, ADP, AMP and IMP as an integral of the signal at approximately 6.14 ppm (Supplementary Fig. S3). The contents of HxR and Hx were obtained from the signals at 6.10 and 8.20 ppm, respectively. Therefore, it was possible to calculate *K* using the ^1^H NMR spectra. Figure 6A shows the *K* values of ribeye samples after different aging durations. *K* reached 1.0 within 4 weeks after starting aging and was unchanged until 10 weeks. Therefore, the long-term aging (over 4 weeks) of ribeye could not be evaluated using *K*. Since the contents of leucine ([Leu]) and creatine ([Cr]) were correlated with long-term aging, the [Leu]/[Cr] ratio is expected to be useful evaluate long-term aging. The [Leu]/[Cr] ratio for aging evaluation was defined as “*L*” in the present study. The muscle tissue structures, such as myofibrillar proteins, are degraded to amino acids including Leu by post-mortem aging^24^. The ^1^H NMR spectra provided the content of leucine and creatine as the integral of the signals at 1.70 and 3.92 ppm, respectively. These signals were derived from two protons of the methylene group (-C***H***_***2***_); therefore, the [Leu]/[Cr] ratio was calculated by dividing the integral value of the signal at 1.70 ppm by that at 3.29 ppm. The NMR-detected [Leu]/[Cr] ratios were plotted against aging duration (Fig. 6B) and showed a strong correlation (*R* ^2^ = 0.844). Larger variance in L was observed in the ribeye samples after longer aging duration, which may reflect the different aging rates among samples.

After slaughtering, the pH of beef markedly decreases and reaches its ultimate pH by the production of lactic acid accompanying glycogen degradation^41^. pH changes have been reported during short-term post-mortem aging (within 3 weeks)^42, 43^. We observed the pH profile of long-term aging (over 4 weeks), which may be used as an indicator of aging progression. Since the D_2_O extract of ribeye contained carnosine, whose imidazole ring is affected by pH^43, 44^, we measured the chemical shift of the ^1^H signal at the C-2 position of the imidazole ring of carnosine [Car H-2] and the pH of ribeye samples with different aging durations. Figure 6C shows a strong correlation between [Car H-2] and pH (*R* ^2^ = 0.984). In addition, the pH decreased with aging progression. These results suggest that the D_2_O extract of ribeye provides the pH profile during aging. However, this pH profile was not consistent with the age-related decrease in lactic acid and may be affected by the buffering power of other components, such as creatine phosphate^45^. The content of acetic acid increased during long-term aging (Fig. 5), which may contribute to the decreased pH profile. pH is related to water-holding capacity, both in raw and heat-treated meats, and tenderness^37^. Therefore, the NMR-detected pH profile may be useful to evaluate meat quality during aging. As compared with regular muscle pH measurements, NMR is useful to measure the pH value under stable conditions such as temperature easily and reproducibly.

## Conclusions

**Figure 7:**
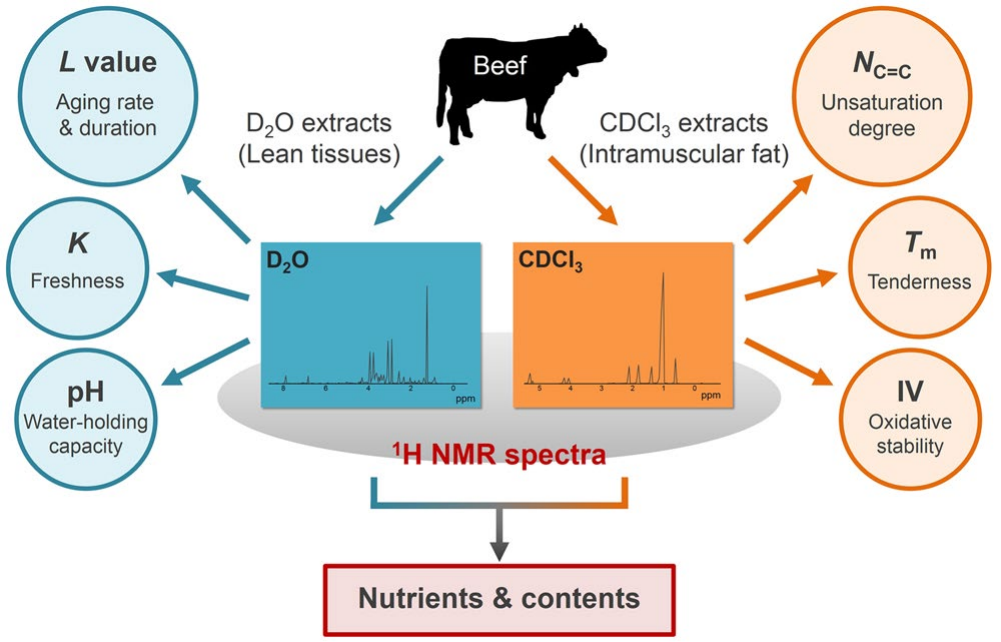
Multiple determinants of primary beef quality that are evaluated by the ^1^H NMR spectra of metabolite extracts from lean tissues and intramuscular fat.

^1^H NMR spectra were applied to evaluate the quality of the lean tissues and intramuscular fat of Japanese Black cattle based on the D_2_O and CDCl_3_ extracts, respectively (Fig. 7). This approach revealed that the NMR-detected unsaturation degree of TAG (*N*_C=C_) was related to the compositional attributes of beef, such as the total ratio of unsaturated fatty acids, melting point, and iodine value (IV). The present study also shows NMR-based metabolic profiles of both polar and non-polar metabolites at longer-term post-mortem aging within 10 weeks. We found that leucine and creatine were available as biomarkers and were, respectively, positively and negatively correlated with aging duration. The *L* value (the ratio of leucine to creatine) was more appropriate to evaluate meat quality during long-term aging than the *K* value. Aging is a common processing technique used to improve meat quality. The ^1^H NMR spectra of the D_2_O extracts may be valuable to monitor the aging process through the detection of the *L* value with other metabolites and the pH profile, which also provides information on meat quality, such as the water-holding capacity and tenderness. This NMR-based metabolomics has potential to evaluate multiple beef qualities detected using different analytical techniques and can be applied to assess other edible meats.

## Materials and Methods

### Beef samples

We obtained beef samples from 29 Japanese Black cattle, which are composed of 25 heifers and 4 delivered cows of four brands in Japan (‘Yamagata beef’, ‘Hida beef’, ‘Sendai beef’, and ‘Kobe beef’) at the ages of 27.7‒39.6 and 103.1‒149.7 months, respectively. All samples were randomly chosen from commercially available dressed carcass at a local meat supplier; therefore, diet and production system were not controlled in the experiments. However, the samples from only a brand are considered to tend to show a similar fatty acid composition. To expand the variation of fatty acid composition, we chose four available brands, though the chosen samples may not be representative of each brand. All Japanese Black cattle are graded using the beef marbling standard (BMS), beef fat standard (BFS), and beef color standard (BCS) by the Japan Meat Grading Association (JMGA). The quality grades of all heifers were higher than Grade 4 (4 or 5). All cattle have been registered in the individual identification information database of the National Livestock Breeding Center, Japan.

### Chemicals

Deuterated water (D_2_O, 99.9%) was purchased from Sigma-Aldrich Co. (St. Louis, Mo). All other regents were supplied from Wako Pure Chemical Industries, Ltd. (Osaka, Japan).

### Sample collection and preparation

Sirloin samples were collected from 5 Japanese Black heifers of 4 brands (total 20 samples). After the storage of slaughtered heifers at 2 °C for 3 days, sirloins were cut and vacuum-sealed. Wet-aging was performed by storing each sample at 2 °C for 2 weeks. Ribeye samples from 5 heifers and 4 delivered cows (‘Yamagata beef’) were treated using the same method as the sirloin samples and were then aged at 2 °C for 2, 4, 6, 8 or 10 weeks (total 42 samples including 2 non-aging samples from delivered cows). Each minced sample (250 mg) was disrupted using a Multi-beads Shocker (Yasui Kikai, Osaka, Japan; metal corn; 2500 rpm for 30 s) with 1.5 ml each of D_2_O and deuterated chloroform (CDCl_3_, 99.8%). The homogenate was centrifuged at 800 *g* for 10 min. The D_2_O and CDCl_3_ phases (each 1 ml) were separately transferred to microtubes and stored at −80 °C until use. The part of CDCl_3_ extracts was dried and weighed for lipid measurement.

### NMR samples

The D_2_O extract (510 *μ*l) was mixed with 130 *μ*l of deuterated sodium phosphate (pH 7.0) and 10 *μ*l of sodium 3-(trimethylsilyl)-1-propane- 1,1,2,2,3,3-*d*_6_-sulfonate (DSS-*d*_6_) at final concentrations of 0.1 M and 34.2 μM, respectively. For the pH evaluation, deuterated sodium phosphate was not added to the D_2_O samples. The CDCl_3_ extract (600 *μ*l) was mixed with 10 *μ*l of hexamethyldisiloxane (HMDS) at a final concentration of 0.45 mM. After centrifugation, the supernatants (600 *μ*L) were transferred to 5-mm NMR tubes.

### NMR spectroscopy

All NMR spectra were measured at 20 °C using a Varian Unity INOVA-600 spectrometer (599.78 MHz for ^1^H and 125.65 MHz for ^13^C) equipped with a cryogenic probe (Agilent Technologies, Ltd., Santa Clara, CA). ^1^H NMR spectra were measured with the following parameters: number of data points, 64 k; spectral width, 7,200 Hz; acquisition time, 4.55 s; delay time, 70.0 s (for D_2_O extracts) or 30.0 s (for CDCl_3_ extracts); and number of scans, 32. The delay time for quantification was determined by the spin-lattice relaxation time (*T*_1_) measurement and was set to >5 × *T*_1_. A ninety degree (90°) pulse was used as the RF pulse to acquire free-induction decay (FID). The water signal was suppressed by the presaturation method.

The parameters for ^1^H-^1^H gradient correlation spectroscopy (gCOSY) were as follows: number of data points, 2,048 (F_1_) and 512 (F_2_); spectral width, 7,200 Hz; acquisition time, 0.155 s; delay time, 2.0 s; and number of scans, 64. The ^1^H-^13^C heteronuclear single quantum coherence (HSQC) was obtained in the phase-sensitive mode with the following parameters: number of data points, 2,048 for ^1^H and 512 for ^13^C; spectral width, 7,200 Hz for ^1^H and 24,128 Hz for ^13^C; acquisition time, 0.155 s; delay time, 7.5 s; and number of scans, 64. The ^1^H-^13^C heteronuclear multiple-bond correlation (HMBC) was measured in absolute mode, and the acquisition parameters were as follows: number of data points, 2,048 for ^1^H and 512 for ^13^C; spectral width, 7,200 Hz for ^1^H and 35,445 Hz for ^13^C; acquisition time, 0.162 s; delay time, 3.5 s; and number of scans, 128.

### Data processing and NMR signal assignment

NMR data were processed using ACD/NMR Workbook Suite V2012 (Advanced Chemistry Development, Inc., Toronto, Canada). Phase and baseline corrections were manually adjusted on the software. The signal assignments were performed by comparison with the published data for beef samples^13, 24^. For the assignment of unreported signals, we predicted candidate compounds using the Biological Magnetic Resonance Bank (BMRB)^46^. Finally, we confirmed that the signals were correctly assigned to the candidate compounds using 2D NMR spectra and spiking experiments^21^. The integral value of each signal was calculated using ACD/NMR Workbook Suite V2012. The integral value of the DSS-*d*_6_ signal at 0 ppm (the trimethylsilyl protons) was used as an internal standard for the quantification of D_2_O extracts.

### GC-FID analysis of the fatty acid composition

Gas chromatography-flame ionization detection (GC-FID) was applied to determine the fatty acid composition of beef tallow. Total lipids were extracted using the Folch method^47^ and were dissolved in benzene. After heating at 80 °C for 20 min with sodium methoxide methanol, methyl-esterified fatty acids were analyzed using a Shimadzu GC-FID 2010 system (Shimadzu Corporation, Kyoto, Japan). Separation was achieved using an InertCap Pure WAX capillary column (30 m × 0.250 mm × 0.25 *μ*m; GL Sciences Inc., Tokyo, Japan) and helium as the carrier gas. The injector and detector temperatures were 240 and 250 °C, respectively. The oven temperature was held at 210 °C for 8 min and was then increased to 230 °C at a rate of 20 °C/min. Finally, the temperature was held at 230 °C for 2 min. The total fatty acid content was calculated by summing the contents of 7 major fatty acids: myristic acid (C14:0), myristoleic acid (C14:1, *cis*-9), palmitic acid (C16:0), palmitoleic acid (C16:1, *cis*-9), stearic acid (C18:0), oleic acid (C18:1, *cis*-9), and linoleic acid (C18:2, *cis*-9,12).

### Melting point measurement of intramuscular fat

The melting point was measured using the ascending heat method employing a glass capillary^48^. Heat-fused intramuscular fat was filled into the glass capillary to a height of 1 cm and was then stored at −30 °C until use. The frozen capillary was placed in a water bath that was initially set to 5 °C. The water temperature was gradually increased using a hot stirrer. When the fused beef tallow rose 1 cm, the temperature was recorded as the melting point.

### pH measurement

The pH of the D_2_O extracts of the beef samples was measured at room temperature before NMR measurements using pH electrodes (HORIBA, Ltd., Kyoto, Japan).

### Statistical data analysis

For the multivariate data analysis, the ^1^H NMR spectra of D_2_O extracts (0.0‒9.0 ppm) were reduced into 0.04 ppm spectral buckets using the ACD/NMR Workbook Suite V2012. The region of the water signal (4.70‒5.15 ppm) was removed, and the sum of spectral buckets was set to 100. Similarly, the ^1^H NMR spectra of the CDCl_3_ extracts (0.5‒5.5 ppm) were reduced into 0.04 ppm spectral buckets and normalized.

The resulting data sets were imported into SIMCA-P version 13 (Umetrics, Umeå, Sweden) as X variables, and Pareto was chosen as the scaling method. Principle component analysis (PCA) was performed as an unsupervised classification method to examine the variation in the data sets. Discriminant analyses based on orthogonal projection to latent structures (OPLS-DA) was used to assess the chemical composition of the ribeye samples with different aging durations, which were imported as Y variables and were scaled with unit variance (UV). The modeled variation in the score plot was defined with a 95% confidence interval. The goodness of fit (*R* ^*2*^) and the predictability (*Q* ^*2*^) described the quality of the OPLS regression model.

Mean values, standard errors and regression lines were calculated using Microsoft Excel 2016 (Microsoft Corp., San Leonardo, CA). Significant differences were analyzed by one-way analysis of variance (ANOVA) using EZR, an interface program of R^49^.

## Electronic supplementary material

Supplementary Information
