# Supplementary material for: NMR-based metabolomics for simultaneously evaluating multiple determinants of primary beef quality in Japanese Black cattle
Source: Sci Rep. 2017 May 2;7:1297. doi: 10.1038/s41598-017-01272-8 (PMC5431055; doi:10.1038/s41598-017-01272-8)
Supplement: Supplementary file 1 — Supplementary Information [file 41598_2017_1272_MOESM1_ESM.pdf]

Supplementary Information

NMR-based metabolomics for simultaneously evaluating multiple determinants of primary beef quality in Japanese Black cattle

Yoshinori Kodani, Takuya Miyakawa, Tomohiko Komatsu, Masaru Tanokura*

*Corresponding author. E-mail: amtanok@mail.ecc.u-tokyo.ac.jp

Contents

Supplementary Table 1: ^{1}H and ^{13}C signal assignments of the compounds detected in D_2O extracts

Supplementary Figure S1. Loading plot for PC2 of the score plot that was generated using the ^{1}H NMR spectra of the D_2O extracts of ribeye samples with different aging duration.

Supplementary Figure S2: PCA score plot of the $CDCl_3$ extracts of ribulose samples with different aging duration (0, 2, 4, 6, 8 and 10 weeks)

Supplementary Figure S3: ^{1}H NMR spectrum of an equimolar mixture of nucleotides (ATP, ADP, AMP and IMP) and HxR

Supplementary Table S1. ^{1}H and ^{13}C signal assignments of the compounds detected in D_2O extracts

Compound	Abbreviation	Assignment	Chemical shift (ppm)	
			^{1}H (multiplicity)	^{13}C
Amino acids				
alanine	Ala	α-**CH**	3.77	53.30
		β-**CH$_3$**	1.48 (d) *	18.89
		**C**OOH		178.60
anserine	Ans	**CH**-2, ring	8.23	139.13
		**C**-4, ring		133.13
		**CH**-5, ring	7.09 (s)	123.27
		N–**CH$_3$**	3.77 (s)	35.93
carnitine		α-**CH$_2$**	2.42	45.72
		β-**CH**	4.57	66.70
		γ-**CH$_2$**	3.42	72.79
		N(**CH$_3$**)$_3$	3.21 (s) *	56.77
		**C**OOH		180.40
carnosine	Car	β-alanyl α-C**H$_2$**	2.67 (m) *	
		β-alanyl β-C**H$_2$**	3.21	
		Histidyl α-C**H**	4.47 (m)	
		Histidyl β-**CH$_2$**	3.09	29.99
		**CH**-2, ring	8.18 (s)	137.08
		**C**-4, ring		133.95
		**CH**-5, ring	7.11 (s)	119.85
		**C**OOH		179.35
		**C**ONH		174.42
creatine	Cr	α-**CH$_2$**	3.92 (s)	56.57
		N–**CH$_3$**	3.03 (s) *	39.65
		**C**OOH		177.43
		NH=**C**(NH$_2$) –N		159.89
glutamic acid	Glu	β-**CH$_2$**	2.05 (m)	29.70
		γ-**CH$_2$**	2.35 (m) *	36.07
		α-CH$_2$–**C**OOH		177.19
		γ-CH$_2$–**C**OOH		183.81
glutamine	Gln	β-C**H$_2$**	2.13 (m)	
		γ-C**H$_2$**	2.45 (m) *	
		**C**OOH		176.74
		**C**ONH$_2$		180.40

(to be continued)

Supplementary Table S1 (continued)

Compound	Abbreviation	Assignment	Chemical shift (ppm)	
			^{1}H (multiplicity)	^{13}C
glycine	Gly	α-CH_2	3.55	44.09
		COOH		175.17
isoleucine	Ile	α-CH	3.66	62.42
		β-CH	1.97	38.69
		γ-CH_2		27.22
		γ'-CH_3	1.00 (d) *	
		δ-CH_3	0.93	13.59
		COOH		177.43
leucine	Leu	α-CH	3.73	56.17
		β-CH_2	1.70 (m) *	42.59
		γ-CH	1.69 (m) *	26.96
		δ-CH_3	0.94	23.68
		δ'-CH_3	0.96	24.78
		COOH		178.35
phenylalanine	Phe	β-CH_2	3.12, 3.27	
		C-1, ring		137.90
		CH-2,6, ring	7.32 (d) *	131.78
		CH-3,5, ring	7.42 (dd)	131.65
		CH-4, ring	7.38 (t)	130.28
tyrosine	Tyr	C-1		129.61
		CH-2,6, ring	7.18 (d)	133.50
		CH-3,5, ring	6.88 (d) *	118.55
		CH-4		157.91
valine	Val	α-CH	3.60 (d) *	63.04
		β-CH	2.27	31.98
		γ-CH_3	0.97 (d)	19.42
		γ'-CH_3	1.03 (d)	20.50
Sugars				
α-glucose	α-Glc	CH-1	5.23 (d) *	94.67
		CH-2	3.53	74.04
		CH-3	3.71	75.30
		CH-4	3.40	72.29
		CH-5	3.83	74.06
		CH_2-6	3.77, 3.84	63.24

(to be continued)

Supplementary Table S1 (continued)

Compound	Abbreviation	Assignment	Chemical shift (ppm)	
			^{1}H (multiplicity)	^{13}C
β-glucose	β-Glc	**CH**-1	4.64 (d) *	98.57
		**CH**-2	3.24	76.84
		**CH**-3	3.47	72.29
		**CH**-4	3.40	72.29
		**CH**-5	3.47	78.55
		$\mathbf{CH_2}$-6	3.72, 3.90	63.42
Purine derivatives				
inosine	HxR	**CH**-1’	6.09 (d) *	91.12
		CH-2	8.23	
		**CH**-8	8.34 (s)	142.88
inosine monophosphate	IMP	CH-1’	6.14 (d) *	
		CH-2	8.22	
		CH-8	8.58 (s)	
hypoxanthine	Hx	CH-2	8.2 (s)	
		CH-8	8.19	
Organic acids				
acetic acid	AcOH	$\mathbf{CH_3}$	1.93 (s) *	25.53
		**C**OOH		183.81
fumaric acid		CH=**CH**	6.51 (s) *	
		**C**OOH		177.38
lactic acid		α-**CH**	4.11 (q)	71.23
		β-$\mathbf{CH_3}$	1.32 (d) *	22.90
		**C**OOH		185.28
Alcohol				
ethanol	EtOH	$\mathbf{CH_3}$	1.17 (t) *	19.45
		$\mathbf{CH_2}$	3.65	60.13
glycerol		$\mathbf{CH_2}$-1	3.55 (m)	65.16
		**CH**-2	3.78 (m)	74.74
		$\mathbf{CH_2}$-3	3.66 (m) *	65.16
Other compounds				
betaine		N($\mathbf{CH_3}$)$_3$	3.25 (s) *	56.17
		$\mathbf{CH_2}$	3.90 (s)	68.93
		**C**OOH		172.02
choline		N($\mathbf{CH_3}$)$_3$	3.19 (s)	56.45

*Proton signals were chosen for the quantitative analysis.

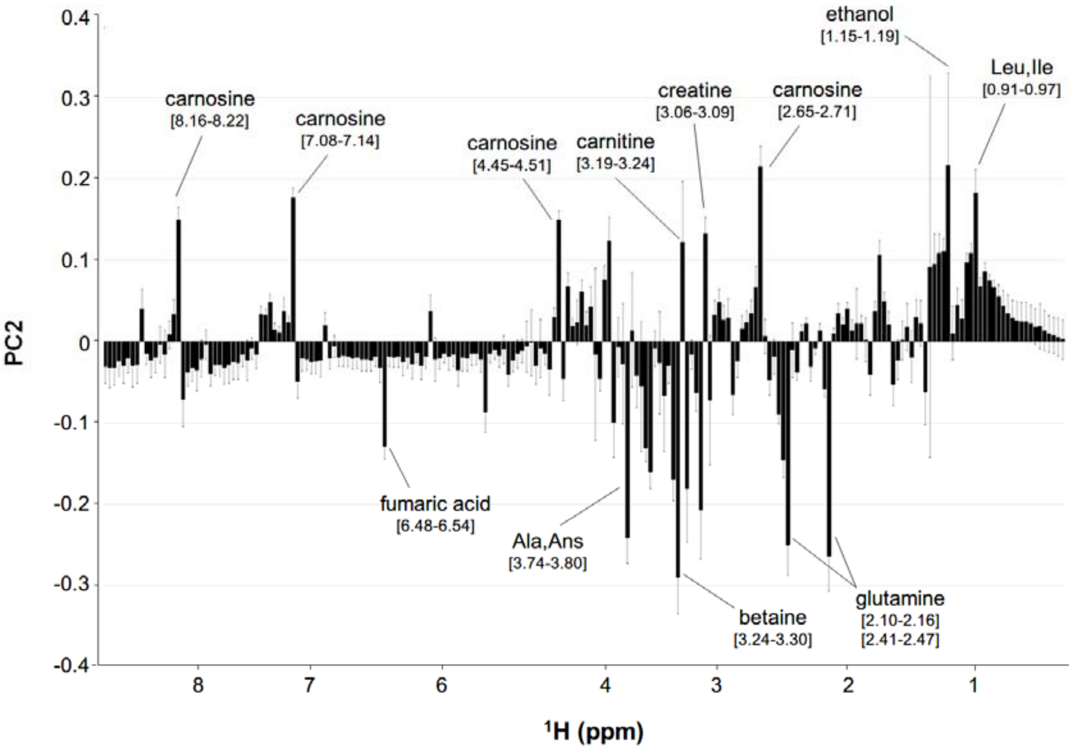

Supplementary Figure S1. Loading plot for PC2 of the score plot that was generated using the ^{1}H NMR spectra of the D_2O extracts of ribeye samples with different aging duration. The PCA score plot is shown in Fig. 4A. Buckets with high loading values are labeled by the assigned compound names. The square brackets represent the chemical shift range of each spectral bucket.

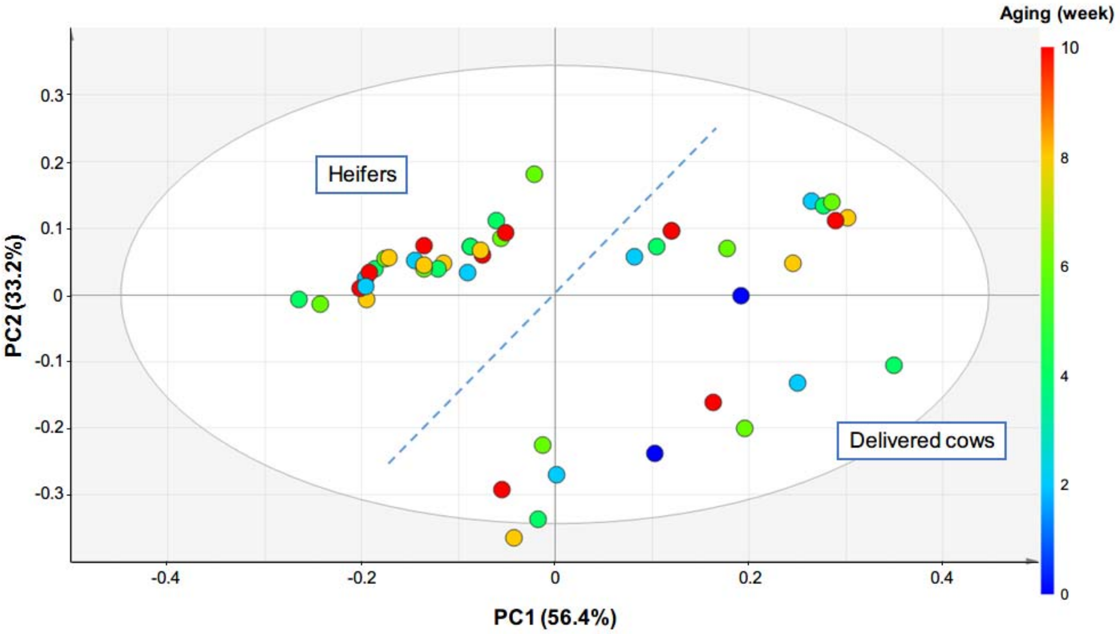

Supplementary Figure S2. PCA score plot of the $CDCl_3$ extracts of ribulose samples with different aging duration (0, 2, 4, 6, 8 and 10 weeks). Ribulose samples are derived from heifers and delivered cows. The heifer samples are separated from the delivered cow in the score plot, and the individual regions are divided by a dashed line.

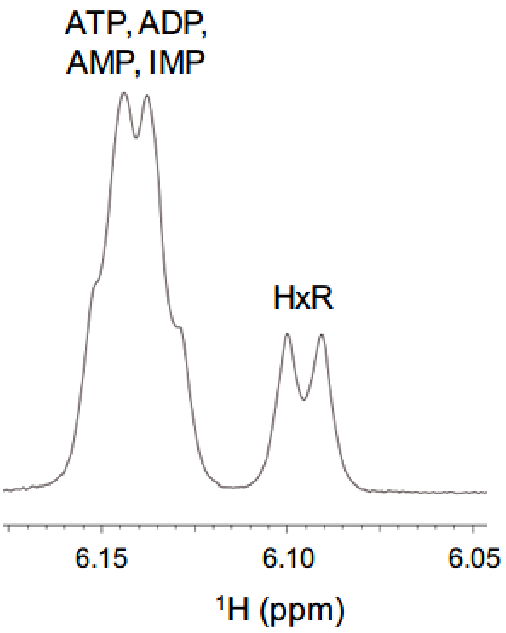

Supplementary Figure S3. ^{1}H NMR spectrum of an equimolar mixture of nucleotides (ATP, ADP, AMP and IMP) and HxR. The signals observed near 6.10 and 6.15 ppm were derived from the ^{1}H attached to the 1’ position of the ribose ring. The nucleotides signals overlapped.
